# The functional organisation of the centromere and kinetochore during meiosis

**DOI:** 10.1016/j.ceb.2025.102486

**Published:** 2025-02-26

**Authors:** Lori B. Koch, Adele L. Marston

**Affiliations:** Centre for Cell Biology, University of Edinburgh, Edinburgh EH9 3BF, United Kingdom

## Abstract

Meiosis generates gametes through a specialised cell cycle that reduces the genome by half. Homologous chromosomes are segregated in meiosis I and sister chromatids are segregated in meiosis II. Centromeres and kinetochores play central roles in instructing this specialised chromosome segregation pattern. Accordingly, kinetochores acquire meiosis-specific modifications. Here we contextualise recent highlights in our understanding of how centromeres and kinetochores direct the sorting of chromosomes into gametes via meiosis.

Centromeres are regions on chromosomes that associate with microtubule fibers during cell division to segregate chromosomes into the new cells. Despite their essential function, centromeres vary widely between organisms. The simplest organisation is the so-called “point” centromere of the budding yeast *Saccharomyces cerevisiae*, where a defined 125bp of DNA sequence is wrapped around a single nucleosome, containing the centromere-specific histone H3 variant Cse4^CENP−A^ upon which the kinetochore assembles and binds a single microtubule [1]. Fission yeast and humans are examples of organisms with ‘regional’ centromeres, consisting of a “core” containing multiple stretches of CENP-A-containing chromatin, surrounded by pericentromeric outer repeats of heterochromatin, in which histones are modified by H3K9 methylation (mono-,di-,and tri-) [1] (Figure 1). Multiple stretches of CENP-A-containing chromatin form the base of compound kinetochore complexes that associate with multiple microtubules at once, around 10e15 in humans [2]. Kinetochores of higher eukaryotes have been proposed to consist of repeating copies of a single kinetochore unit similar to that found in budding yeast [1], however, larger assemblies have yet to be purified or reconstituted and this remains an active area of research. Certain organisms, including various species of plants, insects and the roundworm *Caenorhabditis elegans*, have holocentric chromosomes, which have multiple microtubule attachment sites that can extend over the whole length of the chromosome [3]. It appears that holocentricity evolved from monocentricity multiple times across evolution [4], however, the selective pressures that led to this adaptation remain unclear.

## Meiosis is a unique type of cell division

**Figure 2:**
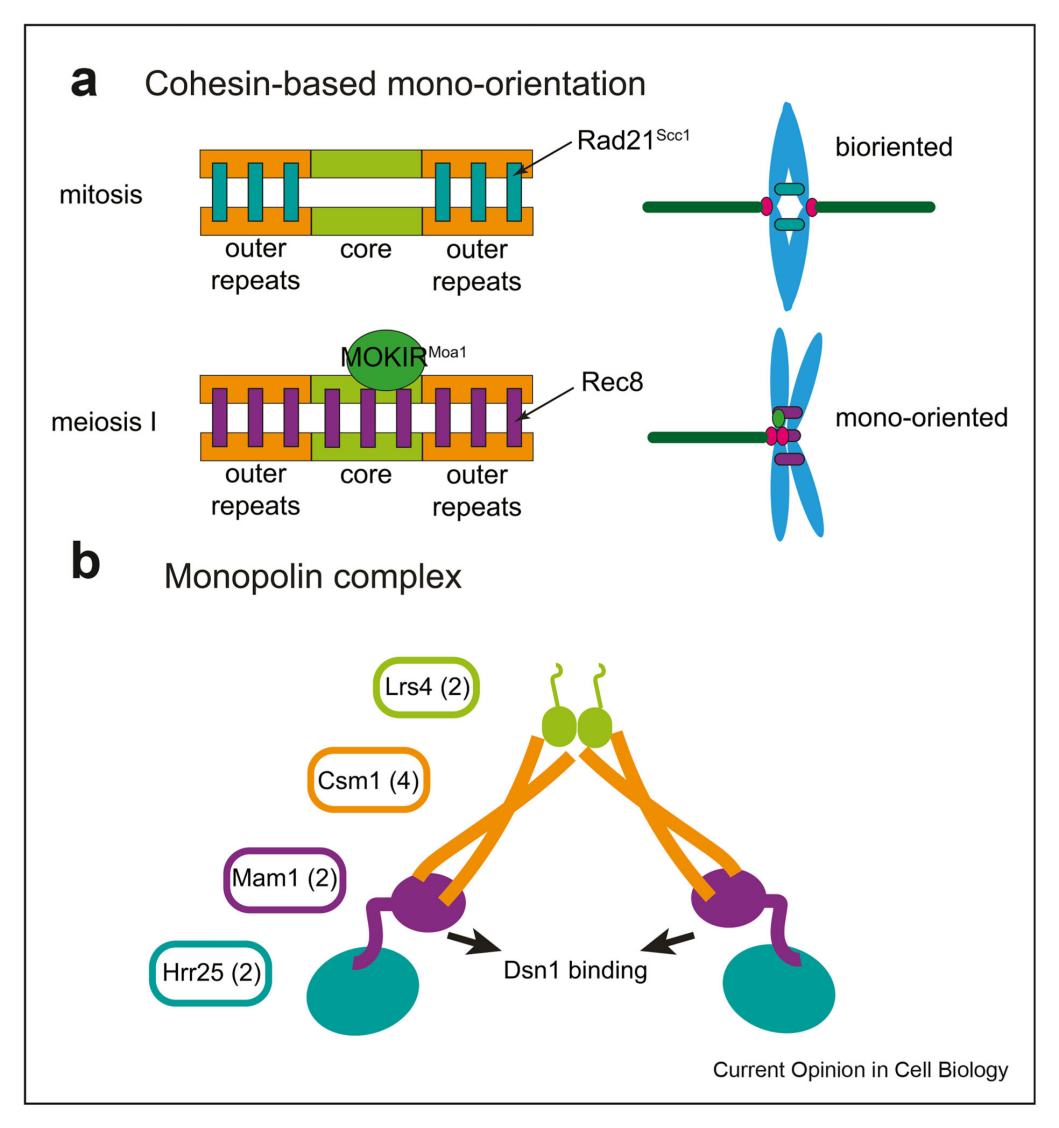
Mechanisms of mono-orientation. **(a)**. Cohesin distribution in the centromeres and pericentromeres could specify the mono-oriented or bioriented state. MOKIR (Moa1/MEIKIN/Spo13) protects core centromere cohesin during meiosis. **(b)**. Model of the budding yeast monopolin complex.

**Figure 3:**
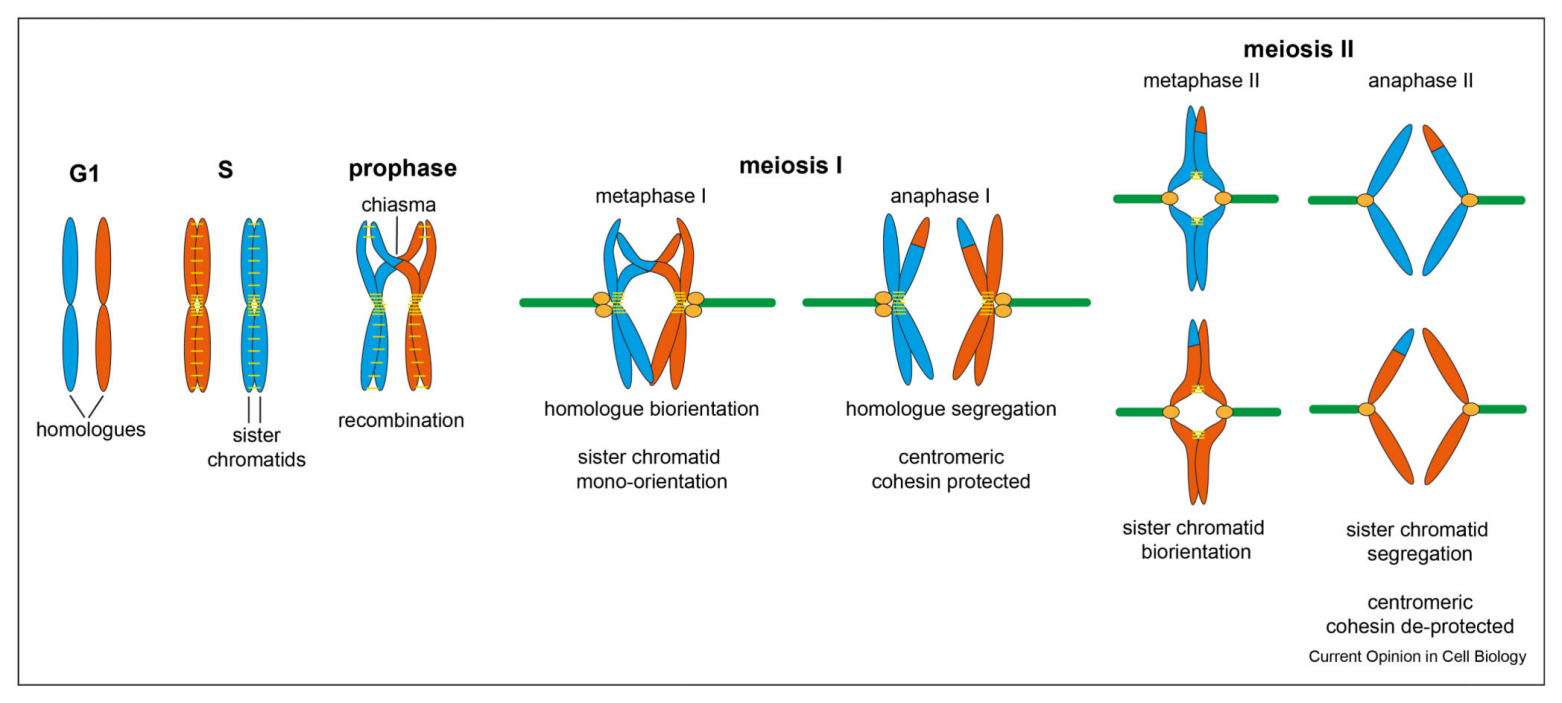
The step-wise loss of cohesin in meiosis. In meiosis I, arm cohesin is lost, while pericentromeric cohesin is protected so that sister chromatids stay together. A small pool of cohesin at centromeres that is implicated in kinetochore mono-orientation is lost in telophase I. In meiosis II, pericentromeric cohesin is cleaved and sister chromatids segregate.

Meiosis is a specialised cell division that generates haploid gametes for sexual reproduction. The reduction in chromosome number is accomplished by two sequential divisions without intervening DNA replication and key differences in chromosome behaviour distinguish meiosis from mitosis [6]. First, in meiotic prophase, DNA double strand breaks (DSBs) initiate recombination between homologous chromosomes [7]. Most organisms rely on crossover recombination and the resultant chiasmata to link homologous chromosomes and allow their biorientation on the meiosis I spindle. In the canonical programme found in budding yeast, mammals and plants, recombination triggers the formation of a large protein structure named the synaptonemal complex (SC), which forms connections between homologues along their entire length, culminating in synapsis. Interestingly, despite their importance, synaptonemal complex proteins are not conserved at the sequence level across evolution [8,9]. Recently, the discovery of homology between kinetochore proteins in the divergent eukaryotic kinetoplastid lineage and synaptonemal complex components has led to the suggestion that kinetochores in these organisms may have evolved from early meiotic SC proteins [10]. A second unique aspect of meiosis is the orientation of chromosomes on the meiosis I spindle. In meiosis I, homologous chromosome pairs biorient on a bipolar spindle and sister chromatids are oriented towards the same pole, called mono-orientation (Figure 2). Thirdly, during mitosis, sister chromatids are segregated in anaphase when the cohesin complex holding them together is cleaved by the enzyme separase. During meiosis, cohesion between sister chromatids must be maintained until they are segregated in meiosis II. Thus, pericentromeric cohesin must be specifically protected from cleavage in meiosis I and then ‘deprotected’ in meiosis II (Figure 3).

### Centromere coupling, synapsis and homologue biorientation

In budding yeast and flies, but not in mice and maize, early meiotic associations between centromeres of non-homologous chromosomes have been observed, a phenomenon that has been called centromere coupling [11]. In budding yeast, coupling depends on the protein Zip1 and in flies C(3)G, both of which are a part of the synaptonemal complex that forms later in prophase [11]. The function of coupling remains largely mysterious, although various explanations have been suggested, including assisting in homology search and preventing recombination at the centromere [11]. Early centromere coupling is dissolved by the beginning of the recombination process. As the homologous chromosomes find each other, synapse and undergo repair, the telomeres associate with the nuclear envelope and the chromosomes rapidly move around. These motions are called ‘rapid prophase movements’ in mammals and in yeast this stage is called the ‘bouquet’ [7]. Genetic studies have demonstrated that disturbing the spatial clustering or prophase movement disrupts homologue synapsis and increases entanglements [7].

Most DSBs are repaired through a non-crossover resolution pathway, and often a single crossover is made between homologue pairs. After DSBs are resolved, the synaptonemal complex disassembles. In budding yeast, oocytes of the fruit fly *Drosophila melanogaster*, and mouse spermatocytes, some synaptonemal complex proteins remain at centromeres, where they mediate associations between homologues that persist until anaphase I [11]. It is thought that these centromere associations promote the correct biorientation and segregation of homologues in meiosis I, and this is especially important for ‘non-exchange’ homologues, which lack a crossover [11,12].

In fruit fly oocytes, correct segregation of achiasmate chromosomes is thought to be mediated by chromosome threads, which link homologues [13]. In fly spermatocytes, where all chromosomes are achiasmate, a unique mechanism occurs whereby homologues are linked by divergent proteins with homology to cohesin [14]. In some Lepidoptera species, such as the silkworm *Bombyx mori*, achiasmate segregation in oocytes is achieved via assembly of a ‘bivalent bridge’, which is assembled from reorganised synaptonemal complex components [15]. Budding yeast can also tolerate one pair of non-exchange chromosomes, which are proposed to associate by process of elimination after the other homologues pair up through conventional recombination-based association [11]. Non-homology-based interactions between chromatin loops, stabilised by cohesin, have been suggested to contribute [16].

The unusual configuration of homologous chromosomes poses a challenge for the segregation machinery, even when linked by crossovers. In budding yeast, efficient capture of homologues onto the meiosis I spindle relies on the engagement of the microtubule regulator Stu1^CLASP^ by the Mad3^BubR1^ spindle checkpoint protein, a pathway that becomes critical for non-exchange chromosomes [17]. Similarly, detailed 4D imaging of holocentric chromosomes in *C. elegans* oocytes identified two redundant mechanisms critical for homologue segregation in meiosis I [18]. The first relies on chromosome pushing by a module comprising BUB1^Bub1^, HCP-1/2^CENP−F^ and CLS-2^CLASP^, while the second requires pulling via the NDC-80 outer kinetochore protein. Therefore, specific requirements for homologue segregation in meiosis I are only just beginning to be uncovered but are an important area for future research.

### Suppression of recombination near centromeres

Crossover formation close to centromeres leads to aneuploidy and is typically suppressed, but the underlying mechanism is not well understood [19]. This ‘centromere effect’, originally discovered in flies, refers to the polar effect of centromeres in preventing crossovers in the surrounding euchromatin [20,21]. Recombination is additionally suppressed within the repetitive pericentromeric heterochromatin flanking centromeres, but this appears to be distinct from the centromere effect [21,22].

Consistently, the centromere-effect is observed even in organisms without heterochromatin, such as budding yeast, where two functions of the Ctf19 kinetochore complex are critical for centromeric crossover suppression [23]. First, Ctf19 prevents DSBs formation over ~ 6 kb around centromeres, which may be due to its requirement for kinetochore integrity [24]. Second, crossover suppression extends to ~ 20 kb surrounding centromeres owing to cohesin recruitment to centromeres by Ctf19, which may favour the repair of residual DSBs from the sister chromatid, rather than the homologue. In support of this idea, crossover formation at an ectopic site was reduced by Ctf19 tethering, but only when it was capable of binding the cohesin loader [25]. In mitosis, Ctf19-driven cohesin loading at centromeres leads to the formation of chromatin loops on each side, and similar loops are also observed in meiosis [26,27]. An attractive hypothesis is that the looped conformation of the pericentromere, which is driven by kinetochore-associated factors, prevents homologue interactions to suppress crossovers.

In fission yeast, centromeres are flanked by repetitive pericentromeric heterochromatin which is associated with cohesin complexes containing the canonical variant of the cohesin subunit Scc3/SA, called Psc3^STAG1/2^. In contrast, chromosome arms are enriched with cohesin complexes containing the meiosis-specific Scc3/SA variant, Rec11^STAG3^ [28]. The exclusion from pericentromeres of Rec11^STAG3^, which interacts with Rec10 and other DSB-promoting factors, prevents recombination [29]. This contrasts with budding yeast, where cohesin suppresses crossovers, but not at the level of DSB inhibition [23]. In the plant *Arabidopsis thaliana*, structural polymorphisms and DNA methylation suppress crossover recombination within the megabase heterochromatic pericentromeres [30]. Whether these effects of cohesin and DNA methylation in fission yeast and *A. thaliana*, respectively, represent true centromere effects or part of the mechanism underlying heterochromatin-based suppression of recombination is unclear. Conversely, whether regional centromeres adopt a looped structure that could contribute to crossover suppression is not known.

### Kinetochore assembly in meiosis

In meiotic prophase, the outer microtubule-binding elements of the budding and fission yeast kinetochores disassemble [31–33]. In budding yeast, this is a consequence of repression of *NDC80* transcription along with degradation of the Ndc80 protein, triggered by Ipl1-dependent phosphorylation [34]. The reason why the outer kinetochore is absent in meiotic but not mitotic prophase is unknown, but one possibility is to ensure that telomeres, rather than centromeres, connect to microtubules to promote the rapid prophase movements of chromosomes [35]. Alternatively, it may facilitate the establishment of a kinetochore that is specialised for meiosis I (see below). Nevertheless, preventing Ndc80 degradation does not prevent meiotic chromosome segregation, so disassembly of the outer kinetochore may not be essential during unperturbed conditions.

Components of the inner kinetochore constitutive centromere-associated network of proteins (CCAN) are essential for kinetochore integrity in budding yeast meiosis, but not mitosis, indicating that there are additional requirements for kinetochore assembly in meiosis vs mitosis [24]. Similarly, in flies, the presence of the CCAN component CENP-C in meiotic prophase is required to ensure proper kinetochore function during the later meiotic divisions [36]. Budding yeast CCAN shows genetic interactions with Aurora B^Ipl1^ [37,38] and CCAN proteins recruit Aurora B^Ipl1^ to kinetochores in mitosis [39,40]. Whether Aurora B^Ipl1^ is also recruited by CCAN in meiosis is unknown, but Aurora B^Ipl1^ is implicated in kinetochore assembly [41–43], and an attractive hypothesis is that CCAN recruited by Aurora B^Ipl1^ plays a specific role in kinetochore assembly in meiosis. Consistently, a different pool of Aurora B^Ipl1^, recruited via Bub1/Bub3/Sgo1, is critical to destabilise kinetochores that are incorrectly attached to microtubules in meiosis I and II [44,45]. Intriguingly, mouse oocytes express a splice variant of the Dsn1 kinetochore component, which bypasses the requirement for Aurora B-dependent phosphorylation for its incorporation into the kinetochore [46], indicating that specialised kinetochore assembly pathways may be a conserved feature of meiosis. Such mechanisms may serve to ensure recruitment of key meiotic regulators at the appropriate time.

### Mono-orientation in meiosis I

Mono-orientation of sister chromatids in meiosis I (Figure 2) requires cohesin in many species and it is thought that a specific pool of cohesin in the core centromere is required to link sister centromeres together to direct their attachment to microtubules from the same pole [27,47–49]. In fission yeast, centromeric cohesion is proposed to constrain sister kinetochores into a side-by-side orientation. Co-segregation of sister chromatids can be enforced in meiosis by artificial tethering of core centromeres [49]. Interestingly, tethering sister centromeres in mitosis by the same method also caused co-segregation of sister chromatids, though to a lesser extent, suggesting meiosis-specific regulation [49]. In flies, a domain analysis of the Spc105R^KNL1^ kinetochore protein revealed the importance of the protein phosphatase I (PP1) binding motif in the N-terminal domain for promoting centromere cohesion and meiosis I mono-orientation [50]. In budding yeast, centromeric cohesion and mono-orientation requires that the cohesin acetyltransferase Eco1 prevents Wpl1 from releasing cohesin from chromosomes [27]. How these factors instruct the establishment and maintenance of centromeric cohesion specifically in meiosis I requires further investigation.

A meiosis-specific regulator of Polo kinase, known as Spo13 in budding yeast, Moa1 in fission yeast and meiosis specific kinetochore protein (MEIKIN) in mammals, collectively referred to as MOKIRs (meiosis one kinase regulator), is crucial for mono-orientation [51]. In budding yeast, phospho-proteomics revealed the global effects of Spo13 on Polo activity and indicated that Spo13 promotes phosphorylation of substrates containing the motif [DEN]x[ST]*F in meiosis I [52]. Thus, it seems likely that Spo13-Polo deposits many different phosphorylations to bring about mono-orientation. Consistently, fission yeast Moa1 was reported to bring about mono-orientation by facilitating Polo^Plo1^ -dependent phosphorylation of cohesin subunits Rec8 and Psc3^STAG1/2^, but the mechanistic consequences of this and whether additional substrates exist are unclear [53,54]. MOKIRs recruit Polo kinase to the kinetochore [55,56] and in budding yeast tethering Polo to the kinetochore is sufficient to promote mono-orientation in the absence of other known mono-orientation factors [55]. In fission yeast, Moa1 localisation depends on CENP-C^Cnp1^ and mutations in CENP-C that disrupt Moa1 localisation also have mono-orientation defects [53,57]. Flies also have a likely MOKIR orthologue, called Matrimony (Mtrm), however, whether it influences mono-orientation is unclear [58]. Nevertheless, like other MOKIRs, Mtrm has broad functions in cell cycle regulation and cohesin protection [51]. How MOKIRs elicit these functions and whether their kinetochore localisation is important is unknown.

Budding yeast additionally have a dedicated fourprotein complex, monopolin, that is required for mono-orientation, consisting of Casein kinase I (CK1), Mam1, Csm1 and Lrs4 [6,59]. Monopolin binds to kinetochores through the central Dsn1 component of the Mtw1/MIND complex [60,61] and its recruitment depends on Polo^Cdc5^ activity as well as MOKIR^Spo13^ [6]. Interestingly, overexpression of Mam1 and Polo^Cdc5^ is sufficient to induce mono-orientation in mitotic cells, and this requires other components of monopolin but not MOKIR^Spo13^ [62]. However, the effect was not very penetrant, suggesting that additional factors stabilise mono-orientation in meiosis.

Monopolin is V-shaped and is proposed to bridge Dsn1 molecules to fuse sister kinetochores [63,64]. Consistently, each pair of meiotic budding yeast sister kinetochores bind a single microtubule [65]. Further support for the fusion model came from the finding that purified meiotic kinetochores attached to a single microtubule *in vitro* can withstand higher forces before rupture than mitotic kinetochores, and this depends on monopolin [66]. Because CK1 kinase activity is required for monoorientation *in vivo* but not for monopolin localisation to kinetochores or increased kinetochore-microtubule attachment strength *in vitro* [61,66,67], it may provide specificity to ensure that sister kinetochores are fused.

It is not clear why a dedicated monopolin complex that facilitates mono-orientation has only been described in budding yeast. It is possible that the difference in centromere organisation calls for distinct mechanisms or perhaps divergent proteins with a similar function to monopolin exist in organisms other than budding yeasts, but have yet to be identified. It is also likely that diverse mechanisms have evolved to orient sister chromatids towards the same pole in meiosis I; in the holocentric pantry moth *Plodia interpunctella*, sister kinetochores are surprisingly not paired, but nevertheless mono-orient [68].

### Step-wise loss of cohesin

Cohesin complexes are loaded and link chromosomes together, establishing cohesion, in S-phase. During mitosis, cleavage of cohesin by separase occurs in a single step, triggering the segregation of bioriented sister chromatids to opposite poles. In meiosis, cohesin is lost from chromosomes in a step-wise manner, from chromosome arms in anaphase I, and only later at pericentromeres in anaphase II. Recent findings in mouse oocytes have further suggested that the centromeric pool that is thought to tether sister kinetochores for monoorientation is lost after arm cohesin but before pericentromeric cohesin in telophase I [47,48]. This leads to a three-step model for cohesin loss (Figure 3): (i) loss of arm cohesin in anaphase I triggers homologue segregation, (ii) loss of centromeric cohesin in telophase I reverses sister kinetochore mono-orientation and (iii) pericentromeric cohesin loss in anaphase II triggers sister chromatid segregation.

In meiosis, the Rad21/Scc1 subunit of cohesin is substituted by its Rec8 homologue, which must be phosphorylated to be cleaved by separase. In budding and fission yeast, while Polo and Dbf4-dependent kinase (DDK) may contribute, the major cohesin kinase promoting cleavage is CK1, while in worms and mouse oocytes, Aurora B/C takes on this role [51,69]. In meiosis I, Rec8 phosphorylation in pericentromeres is prevented by shugoshin proteins, which recruit the phosphatase PP2A-B56^Rts1^ [6]. MOKIRs are also required for cohesin protection, and act to restrain the activity of cohesin kinases, but the underlying mechanism is unclear [55]. In budding yeast, the looped structure of pericentromeres appears important in defining the protected domain since cells deficient in cohesin acetylation fail to establish loop boundaries, resulting in meiosis II non-disjunction [27].

Shugoshin proteins protect sister chromatid cohesion until metaphase II. Budding yeast and flies have a single shugoshin protein, while other organisms have two, some of which have mitotic roles. In mice and humans, Sgo2 is the relevant shugoshin for meiotic cohesin protection. In mice, Sgo2 localises to distinct pools at the centromere and pericentromeres in meiosis I and, unexpectedly, the centromeric pool, rather than the pericentromeric pool appears to be important for cohesion protection [70]. Human oocytes also have two pools of Sgo2, forming centromere ‘cups’ and pericentromeric ‘bridges’ between sister centromeres [71]. Sgo2 in the bridge deteriorates with age, and this could contribute to the high frequency of eggs that are aneuploid in older women [71].

Several models have been put forth to explain the ‘deprotection’ of cohesin in meiosis II. A long-standing model posits that in meiosis II, shugoshin-localised PP2A-B56^Rts1^ is moved away from cohesin subunit Rec8 when the sister chromatids are bioriented by opposing spindle microtubules, leading to the increase in Rec8 phosphorylation, which enables its cleavage [72,73]. However, later work demonstrated the insufficiency of tension in promoting cohesin deprotection [48,74]. In mouse oocytes, a potential inhibitor of PP2A, SET/TAF1beta may contribute to the deprotection of cohesin in meiosis II [75], however, orthologues of this protein in budding yeast are dispensable for meiotic chromosome segregation [76]. More recently, activation of APC^Cdc20^ was found to be required for cohesin deprotection, independent of spindle tension, and in yeast key targets are thought to be Sgo1 and the Mps1 kinase that is important for Sgo1 localisation [48,74,76,77].

Elegant work in mouse oocytes suggested that separase-dependent loss of cohesion between centromeres in late meiosis I, when pericentromeric cohesion is still protected, is required for cohesin deprotection in meiosis II [47,48]. Centromeric Rec8 is a likely sub-strate, but the regulatory mechanisms that delay its loss until late meiosis I, while escaping Sgo2-dependent protection remain to be identified. Furthermore, ectopically-produced Meikin can also be cleaved by separase in mitotically-dividing cultured cells and the introduction of exogenous uncleavable Meikin into mouse oocytes has detrimental effects [78]. Understanding the significance of these findings for deprotection and reversal of kinetochore mono-orientation requires further investigation.

## Conclusions and perspective

Aneuploidy in oocytes is a leading cause of infertility and miscarriage in humans. Therefore, understanding the molecular mechanisms that promote the production of healthy gametes is of primary importance for human health. Recent research on mammalian oocytes including mouse, bovine and human, has begun to identify important factors for this process, many of which were discovered in model organisms. Ongoing research in model organisms will continue to enable identification of essential basic mechanisms.

## Figures and Tables

**Figure 1:**
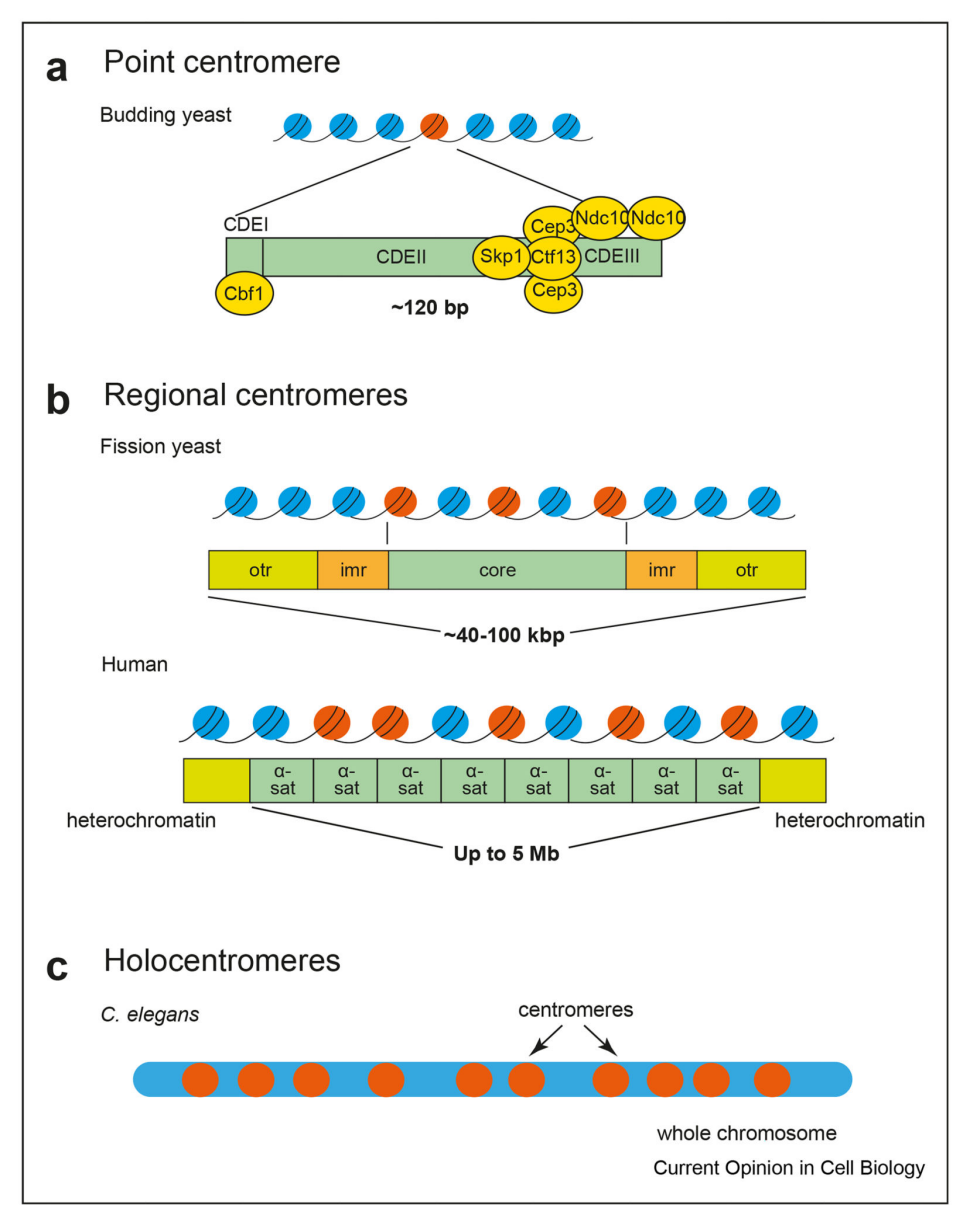
Centromere organisations of different species **(a)**. The point centromere of budding yeast. **(b)**. The regional centromere structure of *S. pombe* and *H. sapiens*. Adapted from Ref. [5]. **(c)**. Holocentric chromosomes have centromeres (represented by orange circles) distributed throughout their length. Diagrams are not to scale.

## Data Availability

No data was used for the research described in the article.
